# Editorial: Through thick and thin - the army of secondary metabolites in plant-fungi interactions

**DOI:** 10.3389/fpls.2024.1374717

**Published:** 2024-03-14

**Authors:** Francesca Degola, Sabrina Palmano

**Affiliations:** ^1^ Department of Chemistry, Life Science and Environmental Sustainability, University of Parma, Parma, Italy; ^2^ Istituto per la Protezione Sostenibile Delle Piante, Consiglio Nazionale Delle Ricerche (IPSP-CNR), Torino, Italy

**Keywords:** plant/fungi interaction, secondary metabolites (SMs), fungal endophytes and pathogens, mycorrhyzae, chemical communication in the plant environment, phytoremediation, fungal metabolism

It is well known that plants and microorganisms have co-evolved complex interactions, often mediated by secondary metabolites, to establish a balance between symbiosis, mutualism, and competition. Less intuitively, other ecological phenomena such as allelopathy and zoophily involve a chemical communication between organisms: for example, some allelochemicals produced by plant root colonizing bacteria and released in the rhizosphere can influence specific interactions of plant species with their environment (Kremer, 2006; Saraf et al., 2014; Kong et al., 2019), whereas microorganisms associated to flowers release volatile compounds that direct pollinators behavior (Rering et al., 2018). In such vast and complex scenario, particular interest plays the interaction between plants and fungi, that communicate through a puzzling chemical language, ruled by small organic compounds that act as crucial mediators in this relationship and possess unique properties and roles. In fact, plants are prone to synthetize a wide array of secondary metabolites (i.e. alkaloids, terpenoids, and phenolic compounds) in response to pathogenic fungi attack, that can directly inhibit the entrance and spread of the enemy in the plant as well as elicit the host’s immune system (Pusztahelyi et al., 2016; Zaynab et al., 2018); on the other hand, fungi counteract with a plethora of different molecules aimed at the plant’s defenses avoidance and/or suppression (Howlett, 2014). However, it should also be considered that pathogenic species usually coexist with endophytic ones, at both aboveground and underground level, that can positively interact with the plant by enhancing nutrient solubilization in the rhizosphere, promoting plant growth or activating plant systemic resistances to biotic or abiotic stresses (Beena et al., 2021).

It is then clear that the plant/fungus communication is more ascribable to a match in which a winner is not expected, but rather a “dialogue” between the two organisms. Volatile organic compounds (VOCs) have been demonstrated to play essential roles in trophic interactions as above as belowground; some of them, that are produced by both soilborne pathogenic and beneficial fungi, are able to influence plant performance or modulate its interaction with the environment. In particular, VOCs from arbuscular mycorrhizal fungi (AMF) can be perceived from a distance by plants and prime their response to microorganisms, promoting at the same time plant growth and tolerance toward biotic and abiotic stress (Duc et al.). Some positive effects of these symbioses are evident in terms of increased biomass and resilience to adverse conditions, such as in the case of increased proline metabolism and nitric oxide accumulation (Liu et al.). However, other possible impacts of AMF on different aspects of the plant fitness have been put under the magnifying glass; for example, the chemical interplay between flowering plants and AMF have been recently reviewed, showing how arbuscular mycorrhizal symbiosis can control -directly or indirectly- the host plants propagative function by providing nutrient, regulating hormone balance, and modulating the production of other metabolites critical for sexual reproduction (Wang and Tang).

Root colonization by AMF is also successfully used to mitigate the detrimental effect of the dramatic increase of soils contamination, mainly due to industrialization, urbanization, mining, fossil fuel burning and agrochemicals input (Sandeep et al., 2019): belowground, the accumulation of heavy metals from anthropogenic activities demonstrated to be possibly reduced using phytoremediation approaches, in which the fungal partner equips the host plant with metabolic compounds that significantly alleviates heavy metal stress by hindering the contaminant uptake or helping in its compartmentalization (Elhamouly et al.).

Besides abiotic stressors, also biotic factors insist on plant environment that might reduce the beneficial effect of AMF colonization on crops, as the secondary metabolites that mediate the plant-fungi mycorrhizal relationship have been found to play a crucial role in the nutrient distribution from a shared resource among different individuals, leading to yield improvement in agricultural and forest systems. The particular case of maize has been highlighted: aboveground insect herbivory, that is considered a major constraint for maize production, showed to not reduce the nitrogen acquisition from soil, but inhibited nitrogen transfer between plants mediated by common mycorrhizal networks (He et al.).

Another particularly intriguing topic in the plant/fungi interaction is the communication between plants and their fungal endophytes: in fact, in contrast to the dynamics typical of plant/pathogen interactions, endophytic fungi have the capacity to establish enduring associations within their hosts that rely on specialized chemical interactions. The existence of this specific, reciprocal exchange of chemical signals have been well characterized between the understory palm (*Astrocaryum sciophilum*), endemic to the northeast region of the Amazon, and its foliar endophytes, by the exploitation of an untargeted UHPLC-MS/MS approach (Pellissier et al.). From a different perspective, the potential of endophytic fungi as elicitors for the biosynthesis of bioactive compounds by their hosts is very fascinating, as it could represent a trump card for possible strategies in medicinal plants quality and yield increase: this possibility has been explored in *Andrographis paniculata*, an herbaceous species commonly used as a traditional medicine in the far East, (Xu et al.) in which, under greenhouse conditions, the main active compounds Andrographolides (ADCs) resulted highly induced by the application of elicitor components obtained from different isolates of *Colletotrichum* sp.

To date, it is undeniable that if many are the enlightened aspects of plant-to-fungi and fungi-to-plant communication, many others are far from being understood; here, exciting new avenues for research have been proposed, that hopefully will contribute to both knowledge and the development of practical strategies for maintaining and enhancing ecosystem resilience to future environmental changes.

## Author contributions

FD: Writing – review & editing, Writing – original draft. SP: Writing – review & editing, Writing – original draft.
